# Physical Activity Increases after an Affectively Arousing Daily Life Event

**DOI:** 10.3389/fpsyg.2017.00518

**Published:** 2017-04-18

**Authors:** Michael H. Pollak, J. Ryan Hart

**Affiliations:** Department of Psychiatry and Behavioral Sciences, Oklahoma State University Center for Health Sciences, TulsaOK, USA

**Keywords:** physical activity, affective states, ambulatory assessment, accelerometry, stress, public speaking

## Abstract

Evidence that structured physical activity can help to regulate affective state has spurred interest in identifying associations between unstructured physical activity and affective states during daily life. The present study examined whether stressful daily life situations that elicit affective arousal also elicit increased physical activity in the form of restless movement. The study compared the physical activity of professors (*n* = 25) after presenting a classroom lecture to their physical activity at the same time of day on a non-lecture workday. The expectation was that lecturing would increase affective arousal, leading to greater restless movement following the lecture compared to the non-lecture control day. The study assessed subjective arousal to confirm that arousal was higher during the lecture. The primary outcome measures were actigraphy-measured standing and stepping times and number of steps and posture transitions. Results indicate that energetic and tense arousal were higher during the lecture than during the control period. Mean (±SE) up time (standing and stepping) for the 1st minute of the 20 minute post-lecture period was double that of the last minute (32.8 ± 5.73 s to 16.5 ± 5.41 s), while it remained low throughout the comparison period on the control day (*p* = 0.01). Subjects also took more steps (*p* = 0.006) and engaged in more transitions between sitting and standing (*p* = 0.02) after the lecture than after the control period. These results support the conclusion that stressful daily life situations that elicit affective arousal also elicit increased physical activity in the form of restless movement and suggest that affective responses to stressful situations may be important determinants of physical activity during daily life.

## Introduction

Laboratory studies find that structured exercise can increase positive affect (Arent et al., 2000; Reed and Ones, 2006; Wipfli et al., 2008; Reed and Buck, 2009) and decrease negative affect (North et al., 1990; Arent et al., 2000; Rethorst et al., 2009). These findings suggest that increasing physical activity levels during daily life might help regulate affective states, thereby alleviating depression and enhancing well-being (North et al., 1990; Stathopoulou et al., 2006; Reed and Buck, 2009; Kanning, 2012). Some have suggested that such regulation might be achieved through increased performance of daily physical activities without adopting structured exercise routines (Schwerdtfeger et al., 2010; Kanning, 2012; Bossmann et al., 2013; von Haaren et al., 2013). This might be of particular importance for those reluctant to initiate or maintain a structured exercise program (Palomo et al., 2008).

Ambulatory studies of associations between momentary affective states and accelerometer-measured physical activity have attempted to demonstrate that physical activity during daily life has a similar effect on affective state during daily life. These studies generally confirm that increases in positive affect or energetic arousal during daily life are preceded by increased bodily movement (Schwerdtfeger et al., 2008; Kanning, 2012; Kanning et al., 2012; Bossmann et al., 2013; Dunton et al., 2014), but find that increases in negative affect or tense arousal during daily life are preceded by either increases (Powell et al., 2009; Kanning, 2012; Kanning et al., 2012) or no change (Schwerdtfeger et al., 2008; Bossmann et al., 2013; von Haaren et al., 2013) in bodily movement, rather than decreases as observed in laboratory studies. These and other studies of similar design also find that bodily movement follows, as well as precedes, affective arousal (Powell et al., 2009; Schwerdtfeger et al., 2010; Dunton et al., 2014). One explanation for the latter finding is that the studies detected co-occurring, rather than sequential, affective arousal and bodily movement. This would be the case if momentary assessments of affective state reflect snapshots of longer duration episodes of affective arousal occurring concomitantly with bodily movement in response to daily life situations. If affective arousal and bodily movement are co-occurring, they might reflect different aspects of an integrated biobehavioral response to arousing or activating life situations, e.g., the fight or flight response (Cannon, 1927), rather than a direct effect of one on the other. This could explain an association between negative affect or tense arousal and bodily movement during daily life.

Cannon (Cannon, 1927) described the fight-or-flight response as an integrated response that mobilizes the body to expend energy via physical activity in response to a threatening situation. If the body prepares to respond to a threatenting situation with physical activity (Cannon, 1927), but then, perhaps because of situational or social constraints, does not actually fight or flee, what happens to the accumulated energy resources? Based on observations that fidgeting tends to occur under conditions that constrain movement, investigators have suggested that fidgeting is a manifestation of an internal or biological urge to move thwarted by situational constraints on movement (Galton, 1885; Mehrabian and Friedman, 1986; Ravussin et al., 1986; Hillemanns et al., 1996). By extension, if individuals experience an urge to move, and are free to move about, they might satisfy this urge with other non-goal directed behavior, such as repeated postural shifting or pacing. Thus, an urge to move, perhaps brought on by the need to expend energy readied, but not expended, in response to a threatening situation, might result in motor restlessness expressed as fidgeting in place under some circumstances and as postural transitions or greater ambulation under other circumstances. At present, the extent to which affect-related body movements during daily life reflect fidgeting, postural transitions, or ambulation is unknown.

That people experience impulses to move or expend energy relates to the concept of arousal. One approach to studying arousal conceptualizes two arousal systems, each of which predisposes individuals to move or to be active, but via different mechanisms (Thayer, 1985, 1989). Thayer (1989) suggested that energetic arousal usually accompanies feelings ranging from energy to tiredness and reflects mobilization of energy for the performance of ordinary behavior during daily life, and that tense arousal usually accompanies feelings ranging from tension to calmness and reflects mobilization of energy in response to perceived threat or danger, i.e., the fight or flight response (Cannon, 1927).

The circumplex model of affect (Russell, 1980) defines affective states in terms of two orthogonal bipolar dimensions labeled affective valence (positive vs. negative) and degree of activation (high vs. low). Energetic and tense arousal can be viewed as rotated dimensions within this model, that is, energetic arousal ranges from activated positive to non-activated negative affect and tense arousal ranges from activated negative to non-activated positive affect (Yik et al., 1999; Ekkekakis et al., 2005).

If both energetic and tense arousal predispose individuals to move, then daily life situations that provoke increases in either type of affective arousal should produce concomitant increases in movement. Indirect support for this contention comes from studies showing that fidgeting occurs under conditions expected to elicit affective arousal, such as a patient waiting in a dentist’s reception area (Barash, 1974), game players waiting for an opponent’s move (Mehrabian and Friedman, 1986), or for an opportunity to win money (Wallace et al., 1975).

Public speaking is a widely studied psychosocial stressor (Dickerson and Kemeny, 2004; Campbell and Ehlert, 2012) which elicits affective arousal, especially tense arousal (Houtman and Bakker, 1987, 1991; Filaire et al., 2010, 2011), and perhaps restless movement. Indirect support for the latter comes from evidence that public speaking tasks in the laboratory (Kirschbaum et al., 1993; Campbell and Ehlert, 2012) and lectures by professors (Filaire et al., 2010, 2011) and student teachers (Houtman and Bakker, 1987, 1991) during daily life elicit affective arousal and sympathetic-adrenomedullary and hypothalamic-pituitary-adrenal axis responses that promote energy mobilization (Kirschbaum et al., 1993; Campbell and Ehlert, 2012). Energy mobilized by public speaking during daily life might be expended as restless movement. We are unaware of any studies that measured physical activity associated with public speaking objectively.

Constraints on movement arising from an individual’s current circumstances might make it difficult to demonstrate that arousing life situations elicit increases in bodily movement during daily life. For example, a particular situation might require a person to sit or stand, or to move about or be still, regardless of the person’s inclination toward movement. For this reason, this study focused on body movement during a period of relative freedom to move following a life event expected to increase affective arousal. We reasoned that, if an increase in arousal in response to a particular life event reflects mobilization of energy in preparation for body movement, but this movement does not occur immediately due to situational or social constraints, this energy might be expended after the event in the form of restless behavior. Other studies have demonstrated prolonged cardiovascular (Pieper et al., 2010) and endocrine and immune (Zoccola et al., 2008) activation after stressful situations.

The present study tests the hypothesis that some stressful daily life events or situations that elicit affective arousal also elicit increases in restless body movement in the form of more standing, more steps in brief episodes, and more frequent postural transitions. The study compares medical school professors’ affective arousal and body movement during and after a regularly scheduled class lecture and at the same time on a non-lecture workday. We predicted that lecturing would increase affective arousal, leading to greater restless body movement following the lecture than during the corresponding time on the non-lecture day. Further, this study provides a more complete picture of affect-related body movement during daily life than most previous studies (Kanning, 2012; Bossmann et al., 2013) by reporting body movement in terms of posture and stepping behavior.

## Materials and Methods

The Oklahoma State University Center for Health Sciences Institutional Review Board approved the protocol for this study prior to recruitment of subjects. All subjects completed a statement of informed consent prior to participation in the study. All experimental procedures were carried out in accordance with the Declaration of Helsinki.

### Subjects

Subjects were 29 volunteers from one medical school faculty who delivered regularly scheduled lectures in 1st or 2nd year medical school classes. All had offices in the medical school building near to the lecture halls. Subjects reported no limitations to daily physical activity nor were they taking medications known to influence motor activity. We did not analyze data from four subjects who left campus or participated in unplanned business meetings during recording periods. Twenty (80%) of the 25 remaining subjects were male. Ages ranged from 41 to 70 years (*M* = 56.2 years, *SD* = 8.4 years). Mean BMI was 26.5 (*SD* = 3.5).

### Measures

#### Posture and Steps

We assessed body movement with a uniaxial accelerometer (activPAL Professional; PAL Technologies LTD) taped unobtrusively to the thigh under clothing. The activPAL samples and stores the acceleration signal at a rate of 10 samples per sec. The device interfaces with a standard personal computer running proprietary software that computes several movement parameters, including sedentary (sitting or lying), standing, and stepping times, step count, and number of sit-to-stand and stand-to-sit transitions. Previous studies show these measures to be valid and reliable (Grant et al., 2006, 2008; Ryan et al., 2006). This study calculated movement parameters using default settings, except that minimum sitting period was changed from 10 to 1 sec.

#### Subjective Arousal

We assessed subjective arousal with the short form of the Activation-Deactivation Adjective Check-list (AD ACL) (Thayer, 1989), a self-report checklist that asks subjects to “describe your feelings *at this moment*” using 20 affect adjectives on a four-point scale ranging from “definitely feel” to “definitely do not feel.” Using standard procedures, we computed scores for two arousal dimensions, Energetic Arousal and Tense Arousal (Thayer, 1989). Scores for each arousal dimension can range from 10 to 40. The AD ACL was especially suited for this study because of its ease of administration, reliable scores that demonstrate construct validity, and items responsive to physical activity interventions (Thayer, 1989). Subjects completed the checklist on diary cards.

### Procedures

During recruitment, subjects provided age, sex, weight, and height and confirmed that they had no limitations to daily physical activity and were taking no medications that influence physical activity.

Subjects participated in the study for approximately 2 h on two different workdays, one that included a lecture and one that did not. Lecture day participation began with a 50-min class period during which subjects delivered a lecture to a class of 1st or 2nd year medical students, followed by an hour of typical work activity. Non-lecture day participation included 2 h of typical work activity, but no lecture, at the same time of day as the lecture day. To minimize the influence of meetings or classes on subject behavior and to make recording periods as similar as possible, subjects selected participation periods during which they expected to engage in usual work activity in and around their offices, but had no scheduled meetings or classes, except for the lecture serving as the potentially arousing event. Monitoring periods began at 9 am (*n* = 12), 10 am (*n* = 11), or 11 am (*n* = 2).

We randomly assigned half of the subjects to complete the lecture day before the non-lecture day. Four of the 25 subjects repeated a monitoring period due to technical issues resulting in data loss or disruption of the lecture by situational factors.

Before each day’s monitoring period, an investigator met the subject at the subject’s office or the subject reported to our laboratory to have an activPAL device taped to his or her thigh in accordance with standard procedures described by the device manufacturer (Pal Technologies Ltd, 2008). During this brief session, subjects answered questions intended to confirm that they were unaware of any situational or health factor that would affect their physical activity during the recording period. We rescheduled subjects who reported a significant factor of this type. Subjects also completed the AD ACL for the first of three times on each day (Time 1) and received instructions to complete the AD ACL again at the end of the lecture (lecture day) or the 1st hour (non-lecture day) (Time 2), and then a third time at the end of the 2nd hour (Time 3). After the session, subjects went about their usual work activities, including lecture during the 1st hour of one monitoring day. Subjects returned the device to the laboratory after each 2-h monitoring period at which time they completed a brief questionnaire asking about start and stop times of the lecture (lecture day only), unanticipated situational or health factors that might have influenced body movement or subjective arousal during the monitoring period, and what they did in general during the monitoring period. For each lecture period, an investigator was present outside of the classroom to confirm lecture beginning and end times.

### Data Analysis

Preliminary analyses showed that Typeday (lecture, non-lecture) ^∗^ Time (beginning of recording period, end of 1st hour, end of 2nd hour for AD ACL scores) and Typeday ^∗^ Minute (means for 20 consecutive minutes for activity variables) effects did not differ significantly by Typeday order, gender, or BMI group, so we excluded these variables from further analyses.

Analyses of AD-ACL scores for Energetic and Tense Arousal consisted of separate repeated measures Typeday ^∗^ Time linear models, followed by contrast analyses comparing each of the first two times to the last time.

For analyses of activPAL data, we divided subjects’ records for each day into two periods. On the lecture day, the first period included the subjects’ lecture time and the second period spanned 20 min after the lecture, starting approximately 5 min after subjects left the lecture hall after the lecture. We determined the moment subjects left the lecture hall by examining each subject’s physical activity record. Since all subjects’ offices were located within a few minutes’ walk of the lecture hall, exclusion of the first 5 min after leaving the lecture hall helped to ensure that the walk back to their office did not confound the results. On the non-lecture day, the two periods for each subject spanned the same times as on that subject’s lecture day.

The activPAL proprietary software identified each accelerometer data point as part of standing, stepping, or sedentary time. The software also counted steps and sit-to-stand and stand-to-sit posture transitions. Since none of the subjects lay down during the recording period, we defined sedentary time as sitting time. We summed standing and stepping time to compute up time, which provides a measure of the time that subjects were not sitting.

To compare mean values between lecture time on the lecture day and the same time on the non-lecture day, we computed separate dependent *t*-tests for each activPAL measure.

To compare the 20-min post-lecture period on the lecture day and the same time on the non-lecture day, for each activPAL measure we computed minute-by-minute sums expressed as seconds per minute. We computed repeated-measures Typeday ^∗^ Minute linear models for mean up time, standing time, stepping time, number of steps, and number of posture transitions. We did not analyze sit time because the results would be redundant with up time. We followed significant Typeday and Typeday ^∗^ Minute effects with tests for linear trends.

## Results

Mean lecture duration was 49.4 (*SD* = 2.5) minutes. During lectures, subjects spent the majority of their time standing (*M* = 95.0%, *SD* = 8.8%), most of the remaining time stepping (*M* = 4.7%, *SD* = 8.2%), and a very small amount of time sitting (*M* = 0.3%, *SD* = 0.89%). Only two subjects’ data profile included sitting during their lecture period. During the first period on the non-lecture day, subjects spent less time standing (*M* = 16.3%, *SD* = 22.9%), *t*(24) = 17.0, *p* < 0.001, a similar percentage of time stepping (*M* = 4.9%, *SD* = 5.1%), *t*(24) = -0.1, *p* > 0.9, and more time sitting (*M* = 78.8%, *SD* = 26.7%), *t*(24) = 14.8, *p* < 0.001.

Mean Energetic Arousal was higher on the lecture day than on the non-lecture day, *F*(1,24) = 4.63, *p* = 0.04, with the largest difference at Time 1, *t*(24) = 2.51, *p* = 0.02, a smaller difference at Time 2, *t*(24) = 1.41, *p* = 0.17, and no difference at Time 3, *t*(24) = 0.04, *p* > 0.9 (see **Table 1**). For mean Tense Arousal, the Typeday ^∗^ Time interaction was significant, *F*(2,48) = 8.49, *p* = 0.001, reflecting higher mean Tense Arousal on the lecture day compared to the non-lecture day at Time 1, *t*(24) = 3.32, *p* = 0.003, and Time 2, *t*(24) = 2.03, *p* = 0.05, but not at Time 3, *t*(24) = -1.79, *p* = 0.09 (see **Table 1**). In addition, on the lecture day, mean Tense Arousal decreased significantly from Time 1 to Time 3, *F*(1,24) = 17.24, *p* < 0.001, and from Time 2 to Time 3, *F*(1,24) = 10.59, *p* = 0.003; during the non-lecture day, mean Tense Arousal did not change significantly over time.

**Table 1:** Affective arousal for both days and for time periods overall and individually.

Time period	Lecture day		Non-lecture day	
	*M*	*SD*	*M*	*SD*
**Energetic arousal**				
Overall^∗^	27.37	3.88	25.84	4.71
Time 1^∗^	28.04	4.45	24.88	6.17
Time 2	27.88	4.92	26.48	5.19
Time 3	26.20	6.62	26.16	5.00
**Tense arousal**				
Overall	19.20	4.05	17.72	5.66
Time 1^∗^	21.36	5.41	17.32	6.27
Time 2^∗^	19.88	4.54	17.40	6.71
Time 3	16.36	5.57	18.44	6.56

*On lecture day, Time 1 is pre-lecture, Time 2 is immediately after lecture, and Time 3 is end of hour after lecture; on non-lecture day, times are beginning of 1st hour, end of 1st hour, and end of 2nd hour, respectively. ^∗^Indicates *p* < 0.05 for lecture day compared to non-lecture day.*

**FIGURE 1:**
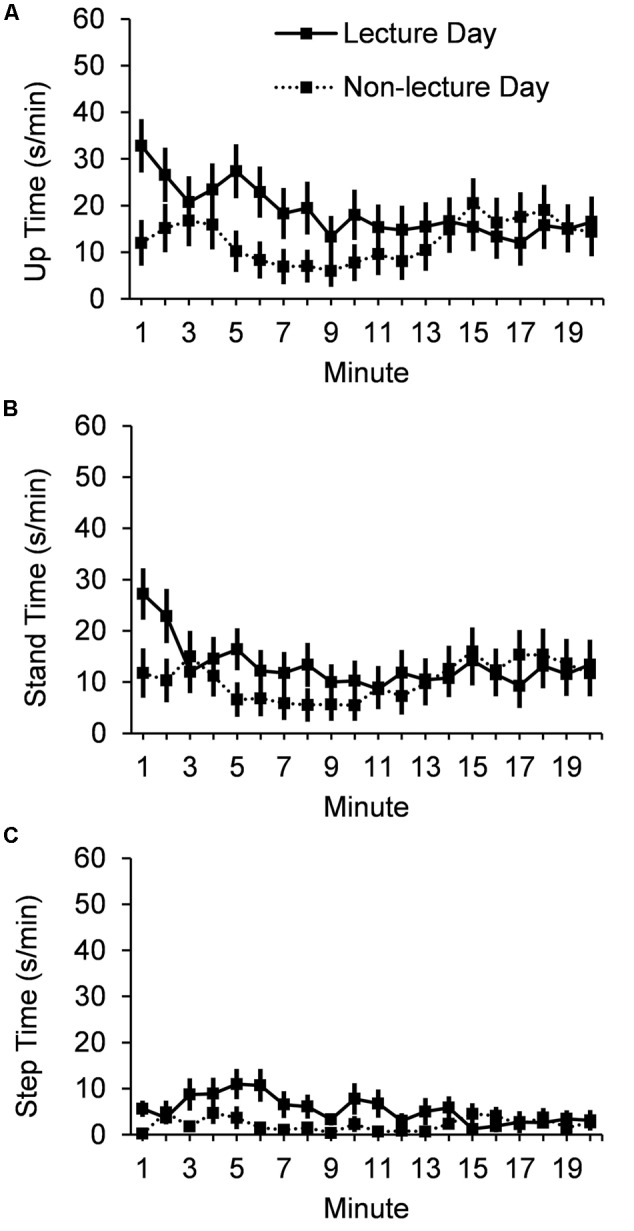
**Mean seconds per minute for 20 min after lecture or control period for (A)** up time, **(B)** stand time, and **(C)** step time. Error bars show standard errors.

A significant Typeday ^∗^ Minute linear trend interaction, *F*(1,24) = 4.9, *p* = 0.04, indicates that up time duration (mean ± SE) (**Figure 1A**) decreased from the first (32.8 ± 5.73 s) to the last (16.5 ± 5.41 s) minute of the 20 min period following the lecture, but showed no difference during the 20 min control period on the non-lecture day (12.0 ± 4.90 s to 14.4 ± 5.23 s). Results for the two components of up time show that mean stand time (**Figure 1B**) did not vary by Typeday (13.3 ± 2.86 s/min vs. 10.3 ± 3.22 s/min), *F* < 1; but that mean stepping time (**Figure 1C**) did vary by Typeday (5.40 ± 1.04 s/min vs. 2.26 ± 0.65 s/min), *F*(1,24) = 7.19, *p* = 0.01.

**FIGURE 2:**
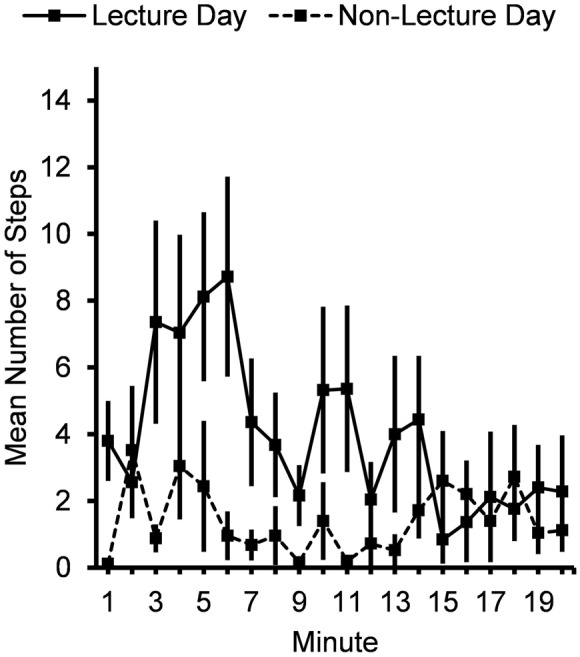
**Mean number of steps per minute for 20 min after lecture or control period.** Error bars show standard errors.

Most stepping episodes following the lecture were brief: the median duration of a stepping episode was 11.0 sec and 75% of stepping episodes lasted no more than 26.0 sec. Subjects also took more steps (**Figure 2**) following the lecture period (4.0 ± 0.78 steps/min vs. 1.4 ± 0.41 steps/min), *F*(1,24) = 9.11, *p* = 0.006.

**FIGURE 3:**
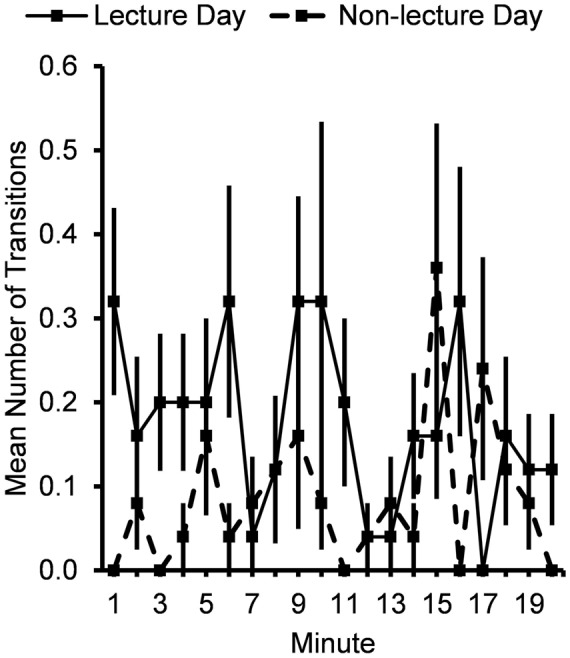
**Mean number of posture transitions per minute for 20 min after lecture or control period.** Error bars show standard errors.

Subjects engaged in more total posture transitions (**Figure 3**) following the lecture period on the lecture day (0.18 ± 0.03 transitions/min) compared to the non-lecture day (0.09 ± 0.02 transitions/min), *F*(1,24) = 6.40, *p* = 0.02, for Typeday main effect, with greater differences at the beginning than the end of the time period, *F*(1,24) = 5.33, *p* = 0.03.

## Discussion

Subjects reported increases in both energetic and tense arousal associated with presenting a lecture in a classroom. After the lecture, subjects stood for more time, took more steps, and exhibited more posture transitions than during a comparable time on a different day preceded by lower energetic and tense arousal. After approximately 15–20 min, these effects had dissipated. To our knowledge, this is the first study to examine changes in objectively measured body movement after an arousing life event. Other studies have reported correlations between momentary affect and body movement levels during daily life, but did not relate these changes to specific life events or situations (Schwerdtfeger et al., 2010; Kanning, 2012; Bossmann et al., 2013).

Our results support the hypothesis that body movement increases after an arousing life event and, thus, that some concomitant increases in subjective arousal and physical activity during daily life are components of an integrated biobehavioral response to arousing or activating life situations, rather than the direct effect of physical activity upon affective state. Our results demonstrate the importance of excluding other potential causes before attributing variations in affective state during daily life to concomitant physical activity. Devices, such as the activPAL, capable of measuring patterns of postural variation and stepping behavior might help to differentiate between exercise-like behavior and other body movements.

Affective arousal on the lecture day was highest before the lecture and lowest at the end of the study period. The decrease in arousal to the approximate levels of the non-lecture day makes it unlikely that the between-day differences in arousal reflected a general difference between days. Similarly, the post-lecture linear trend for body movement makes it unlikely that the between-day differences in body movement reflect a general difference between days. The small number of steps taken during the lecture makes it unlikely that the increase in affective arousal during the lecture was due to ambulation (Schwerdtfeger et al., 2010).

At the end of the lecture, tense arousal remained elevated. This is consistent with the results of other studies of lecturing during daily life (Houtman and Bakker, 1987, 1991; Filaire et al., 2011) and of public speaking tasks in the laboratory (Yamakawa et al., 2009; Balodis et al., 2010). If tense arousal reflects mobilization of energy in response to perceived threat or danger (Thayer, 1989) then the tense arousal seen here might have been elicited by the evaluative threat inherent in public speaking situations (Houtman and Bakker, 1987; Dickerson and Kemeny, 2004).

The role of energetic arousal in the response to public speaking is less certain. Studies that examined changes in energetic arousal associated with public speaking tasks in the laboratory report mixed results, i.e., higher (Carpenter et al., 2011), lower (Balodis et al., 2010), and no change (Yamakawa et al., 2009). In the present study, energetic arousal was higher before the lecture. At the end of the lecture, energetic arousal was no longer significantly higher than at the corresponding time on the non-lecture day, but the linear decrease in energetic arousal from the beginning to the end of the study suggests that energetic arousal had not returned to baseline by the end of the lecture. This consideration makes it difficult to exclude energetic arousal as a potential contributor to the increase in post-lecture body movement. If energetic arousal reflects mobilization of energy for the performance of ordinary behavior during daily life (Thayer, 1989), then the inconsistent results across studies might be related to variability in physical and mental effort (Peters et al., 1998) required by the public speaking tasks in different studies. In the present study, the effort to present nuanced material to a large class in a large lecture hall and maintain psychological focus for an extended time might have elicited greater effort than in most other studies (Dickerson and Kemeny, 2004), which typically involve speaking for a few minutes to a camera or a few other people in a relatively small room.

Although we have no information about the activities of these subjects during the approximately 20 min following the lecture, except that they stayed on campus and did not participate in meetings, during this time they behaved in a way that produced more posture transitions, less sitting, and more steps taken in very brief bouts. This is consistent with an increase in restless non-goal directed movement, which could have occurred while subjects were performing their usual work activities. Restless movement or fidgeting is viewed usually as being motivated by an internal or biological urge to move (Galton, 1885; Ravussin et al., 1986; Hillemanns et al., 1996). In this situation, the most likely source of that urge would be a need to expend energy mobilized by the increase in arousal elicited by the lecture. There are at least three potential explanations for energy mobilized during the lecture to be expended after the lecture. First, persistent thoughts or ruminations concerning the lecture may have prolonged affective arousal and the accompanying mobilization and expenditure of energy. In support of this explanation, laboratory speech tasks that include a component of social-evaluative threat elicit post-task rumination and rumination after such tasks is accompanied by cortisol responses that promote energy mobilization and expenditure after the lecture (Zoccola et al., 2008; Zoccola and Dickerson, 2012). The current study did not assess these subjects’ perception of social-evaluative threat, but it seems reasonable that a lecture delivered to a class of medical students could represent a social-evaluative threat sufficient to elicit ruminative thoughts after the lecture, and that these thoughts could contribute to prolonged affective arousal.

Second, energy mobilized in anticipation of an event may not be well calibrated to need (Obrist, 1981). In this case, energy mobilized in excess of need during the lecture may have been expended after the lecture. This explanation is in line with the idea that increased body movement after the lecture is related to constraints on movement during the lecture.

Third, energy mobilization related to aspects of the physiological response to public speaking, such as increased cortisol secretion, might have a relatively long latency or duration, resulting in energy mobilization after the end of the eliciting situation (Kirschbaum et al., 1993; van Eck et al., 1996).

Future research should focus on determining the extent to which variations in movement levels reflect responses to arousing situations during daily life. Public speaking *per se* may be unlikely to influence behavior in the large majority of adults whose jobs do not require public speaking; however, the characteristics of speaker and situation salient for the elicitation of the responses observed here may occur quite frequently in the daily life of many people. For example, situations that elicit hypothalamic-pituitary-adrenal axis and sympathetic-adrenomedullary responses, such as those occurring during public speaking, tend to be novel or unpredictable, uncontrollable, challenging, or threatening (Mason, 1968a,b; Tomaka et al., 1993; Dickerson and Kemeny, 2004). In particular, cortisol responses during public speaking seem to be related to social evaluative threat and anticipation of uncontrollable outcomes (Dickerson and Kemeny, 2004). Further research should focus on determining the extent to which these factors elicit body movement during daily life.

The unusual level of experimental control exerted over potential sources of variance common to most ambulatory studies strengthens this study. All of the subjects were professors in the same college lecturing in one of two lecture halls to either the 1st or 2nd year class of a single medical school. All of the subjects had offices or labs near to the lecture halls in the same building complex, which means that, after the lecture, they walked to their offices or labs within a few minutes without going outside. We excluded this period of required walking from the analyses. During the post-lecture recording period and the comparable period on the non-lecture day, none of the subjects participated in meetings that would have limited their freedom to sit, stand, or step. The use of a comparison period on a different day controlled for time of day effects and for individual differences in daily affect and activity levels.

While it may have improved internal validity, the control over potential sources of variance may limit the generalizability of these results, e.g., sample size was small and all subjects worked at one educational institution. In addition, subjects selected lecture and non-lecture periods, not only to meet study criteria, but also for convenience. It is possible that these subjects selected atypical times during which to participate in this study; however, they were not aware of the hypothesized relationship between affective state and physical activity, so there is little reason to suspect that their choices contributed to systematic bias in these results. In the same vein, there is little reason to suspect that subject characteristics, e.g., physical fitness or behaviors, e.g., caffeine use, that can influence physical activity during daily life differed systematically between lecture and non-lecture days.

## Author Contributions

MP contributed to the conception and design of the study, analyzed and interpreted the data, wrote initial and subsequent drafts of the paper, and did a final approval. JH contributed to the conception and design of the study, analyzed and interpreted the data, revised the paper critically for important intellectual content, and did a final approval.

## Conflict of Interest Statement

The authors declare that the research was conducted in the absence of any commercial or financial relationships that could be construed as a potential conflict of interest. The reviewer MKL and handling Editor declared their shared affiliation, and the handling Editor states that the process nevertheless met the standards of a fair and objective review.
